# MVA-Based H5N1 Vaccine Affords Cross-Clade Protection in Mice against Influenza A/H5N1 Viruses at Low Doses and after Single Immunization

**DOI:** 10.1371/journal.pone.0007790

**Published:** 2009-11-12

**Authors:** Joost H. C. M. Kreijtz, Yasemin Suezer, Gerrie de Mutsert, Geert van Amerongen, Astrid Schwantes, Judith M. A. van den Brand, Ron A. M. Fouchier, Johannes Löwer, Albert D. M. E. Osterhaus, Gerd Sutter, Guus F. Rimmelzwaan

**Affiliations:** 1 Department of Virology, Erasmus Medical Center, Rotterdam, The Netherlands; 2 Paul-Ehrlich-Institut, Langen, Germany; 3 Ludwig-Maximilians-Universität, München, Germany; University of Georgia, United States of America

## Abstract

Human infections with highly pathogenic avian influenza viruses of the H5N1 subtype, frequently reported since 2003, result in high morbidity and mortality. It is feared that these viruses become pandemic, therefore the development of safe and effective vaccines is desirable. MVA-based H5N1 vaccines already proved to be effective when two immunizations with high doses were used. Dose-sparing strategies would increase the number of people that can be vaccinated when the amount of vaccine preparations that can be produced is limited. Furthermore, protective immunity is induced ideally after a single immunization. Therefore the minimal requirements for induction of protective immunity with a MVA-based H5N1 vaccine were assessed in mice. To this end, mice were vaccinated once or twice with descending doses of a recombinant MVA expressing the HA gene of influenza virus A/Vietnam/1194/04. The protective efficacy was determined after challenge infection with the homologous clade 1 virus and a heterologous virus derived from clade 2.1, A/Indonesia/5/05 by assessing weight loss, virus replication and histopathological changes. It was concluded that MVA-based vaccines allowed significant dose-sparing and afford cross-clade protection, also after a single immunization, which are favorable properties for an H5N1 vaccine candidate.

## Introduction

Over 400 human cases of infections with highly pathogenic avian influenza (HPAI) viruses of the H5N1 subtype have been reported since 2003. More than 60% of these cases had a fatal outcome and new cases continue to be reported frequently[1]. Once these viruses become transmittable from human-to-human by adaption to their new host, a new influenza pandemic is imminent. Neutralizing antibodies against H5N1 viruses are virtually absent in the human population and already nine different clades of antigenically distinct viruses have been identified [2]. Therefore, the development of safe and effective vaccines that, ideally, induce cross-clade immunity has high priority [2]–[4]. The implementation of reverse genetics for the generation of vaccine strains and cell culture technology contribute to the rapid availability of pandemic influenza vaccines [5]–[14]. In addition, the use of adjuvants can increase the immunogenicity of seasonal and pandemic influenza vaccines and may lower the amount of antigen needed for the induction of protective antibody responses [15]–[19].

The development of alternative novel generations of influenza vaccines may mitigate the envisaged shortage of vaccine doses in the future. For example, vector vaccines based on recombinant adenovirus or poxvirus expressing selected influenza virus genes have been shown to be immunogenic and to afford protection against infection with H5N1 virus in animal models [20]–[26].

Especially the replication-deficient modified vaccinia virus Ankara (MVA), constitutes an attractive vaccine production platform. This virus was originally developed as a vaccine against smallpox and has been administered to >120.000 humans without significant side effects [27]. In addition, administration of MVA to immunocompromised individuals is safe and does not lead to systemic disease often associated with the application of replicating vaccinia virus [28], [29]. Its potential as vaccine candidate has been demonstrated for a number of infectious pathogens [30]–[33]. Recently, we have demonstrated that immunization with a recombinant MVA expressing the HA gene of influenza H5N1 virus A/Vietnam/1194/04 (MVA-HA-VN/04) induced protective immunity against infection with the homologous and a heterologous antigenically distinct virus in mice and macaques [24], [25]. In these studies animals were immunized twice with relative high doses (>10^8^ pfu) of recombinant MVA. However, to stretch the number of individuals that can be vaccinated with any given amount of vaccine preparation that can be produced it would be desirable if dose-sparing can be achieved. Furthermore, when a pandemic is imminent, there might not be enough time to induce protective immunity with a two-dose immunization regimen. Thus, ideally, protective immunity is induced after immunization with lower doses and preferable after a single immunization, which are key elements in the development of pandemic influenza vaccines. In the present study, we determined the minimal requirements for the induction of protective immunity with MVA-HA-VN/04 against the homologous virus and against an antigenically distinct H5N1 strain.

Two immunizations with MVA-HA-VN/04 at doses 10,000-fold lower than used previously [25] significantly reduced weight loss and mortality caused by challenge infection with influenza viruses A/Vietnam/1194/04 (clade 1) and A/Indonesia/5/05 (clade 2.1). Strikingly, also protection against the development of clinical signs and mortality was achieved with a single immunization with 10^5^ pfu of MVA-HA-VN/04. The clinical protection correlated with a reduction of virus replication and lung pathology.

Thus, apart from the favorable properties already attributed to recombinant MVA [33], the possibilities of dose sparing and single shot immunization regimens make this vector even more attractive as a pandemic influenza vaccine candidate.

## Results

### Antibody Responses Induced by Immunization with MVA-HA-VN/04

After a single immunization with MVA-HA-VN/04, only mice that received a dose of 10^6^ or 10^8^ pfu developed detectable antibody titers (Table 1). Four weeks after immunization these animals had HI geometric mean titers (GMT) of 6.8 (SD = 1.8) and 15.7 (SD = 2.6) against the homologous virus (A/VN/1194/04) and 5.5 (SD = 1.4) and 6.1 (SD = 1.8) against the heterologous virus (A/IND/5/05), respectively. As shown in table 1, also virus-neutralizing antibodies were detected after a single immunization with 10^6^ or 10^8^ pfu with GMT 5.4 (SD = 1.4) and 5.7 (SD = 1.5) against the homologous strain respectively. Only mice immunized with 10^8^ pfu of MVA-HA-VN/04 developed virus neutralizing antibody titers against the heterologous strain. Mice that received two immunizations with 10^3^, 10^4^, 10^5^ or 10^6^ pfu of MVA-HA-VN/04 developed HI GMT of 6.3 (SD = 2.1), 16.2 (SD = 3.8), 77.1 (SD = 4.2) and 71.9 (3.3) respectively against the homologous strain. Those that received 10^5^ and 10^6^ pfu also developed detectable HI antibodies against the influenza virus A/IND/5/05 with GMT 7.7 (SD = 2.6) and 7.7 (SD = 2.3). In the VN assay, antibodies against the homologous strain were detected with GMT of 5.5 (SD = 1.6), 18.0 (SD = 4.4) and 15.2 (SD = 3.9) in mice immunized with 10^4^, 10^5^ or 10^6^ pfu of MVA-HA-VN/04, respectively. The mice that were immunized twice with 10^5^ and 10^6^ pfu developed virus-neutralizing antibodies against the heterologous strain with GMT of 7.5 (SD = 2.9) and 6.2 (SD = 2.0).

**Table 1:** Induction of antibodies to A/VN/1194/04 and A/IND/5/05 after immunization with MVA-HA-VN/04.

Number of vaccinations	Dose MVA-HA-VN/04(1)	Assay	A/VN/1194/04	A/IND/5/05
One	10^6^	HI	6.8 (1.8)(1)	5.5 (1.4)
	10^8^		15.7 (2.6)	6.1 (1.8)
	10^6^	VN	5.4 (1.4)	-
	10^8^		5.7 (1.5)	5.4 (1.4)
Two	10^3^	HI	6.3 (2.1)	-
	10^4^		16.2 (3.8)	-
	10^5^		77.1 (4.2)	7.7 (2.6)
	10^6^		71.9 (3.3)	7.7 (2.3)
	10^3^	VN	-	-
	10^4^		5.5 (1.6)	-
	10^5^		18.0 (4.4)	7.5 (2.9)
	10^6^		15.2 (3.9)	6.2 (2.0)

(1) titers are expressed as GMT (Standard deviation).

### Protection against Clinical Signs after Infection with Influenza A/H5N1 Virus

From two to three days p.i. onwards, unprotected control animals started to develop clinical signs, irrespective of the challenge virus that was used, although infection with influenza A/IND/5/05 caused more severe disease.

Mice that received PBS or wtMVA once or twice displayed reduced muscle strength, and around day 4 p.i. hunched back posture and heavy breathing. A similar clinical presentation was observed in mice that received one or two immunizations with 10^3^ pfu MVA-HA-VN/04 and that were subsequently infected with influenza virus A/IND/5/05. These mice eventually succumbed from infection or had to be taken out of the experiment because they reached humane endpoints.

Mice that received a single immunization with 10^4^ or 10^5^ pfu MVA-HA-VN/04, and those vaccinated twice with 10^3^ pfu, developed mild clinical signs after infection with the homologous influenza virus A/VN/1194/04. Also mice vaccinated twice with 10^4^ pfu but infected with the heterologous strain A/IND/5/05 had a mild clinical outcome of infection and recovered from infection.

Mice vaccinated once with 10^6^ or 10^8^ pfu and those vaccinated twice with 10^5^ or 10^6^ pfu did not show any clinical signs after infection regardless the virus that was used for infection.

**Figure 1:**
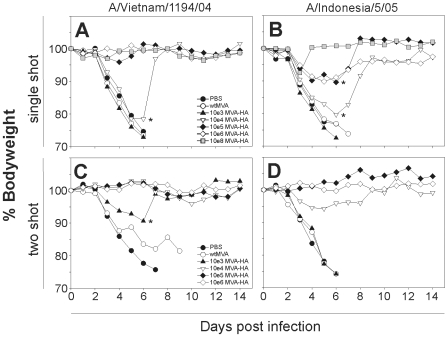
MVA-HA-VN/04 vaccination prevents loss of body weight caused by infection. The bodyweight after infection with influenza virus A/Vietnam/1194/04 (A, C) and influenza virus A/Indonesia/5/05 (B, D) is shown relative to the body weight before infection(100%). Animals were infected four weeks after a single immunization (A, B) with: PBS, wtMVA, 10^3^, 10^4^, 10^5^, 10^6^ or 10^8^ pfu of MVA-HA-VN/04. A second group of animals was infected four weeks after two immunizations (C, D) with PBS, wtMVA, 10^3^, 10^4^, 10^5^ or 10^6^ pfu of MVA-HA-VN/04. (*Indicates weight loss of the proportion of animals in this group that survived post day 6 infection).

In general, the severity of the clinical signs correlated with the extent of weight loss. Mice vaccinated once with doses >10^5^ pfu did not loose weight after infection with influenza virus A/VN/04 and fully recovered (Figure 1). After challenge infection with A/IND/5/05 some weight loss was observed, but it was limited considerably compared to control mice or those vaccinated with doses of <10^5^ pfu MVA-HA-VN/04. Two vaccinations with doses as low as 10^4^ pfu of MVA-HA-VN/04 fully protected mice from weight loss after infection with the homologous strain. Even two vaccinations with 10^3^ pfu prevented severe weight loss observed in PBS control mice and those vaccinated with 10^8^ pfu of the empty vector. Two vaccinations with doses >10^4^ pfu also protected mice from severe weight loss after infection with influenza virus A/IND/5/05

### Survival after Infection with Influenza A/H5N1 Virus

Mice that developed severe clinical signs after H5N1 infection and that showed weight loss of more than 20% were euthanized for ethical reasons. Mice that received a single shot of PBS, wtMVA or the lowest dose of MVA-HA-VN/04 (10^3^ pfu) did not survive past day 7 p.i. with influenza virus A/VN/1194/04 and A/IND/5/05, and most of them reached humane endpoints six days p.i. (Table 2). Mice immunized once with 10^4^ pfu MVA-HA-VN/04 had a survival rate of 25% after infection with both the homologous and heterologous virus. A single vaccination with 10^5^ pfu MVA-HA-VN/04 resulted in 100% survival after infection with the homologous virus and 50% after infection with influenza virus A/IND/5/05. A single vaccination with a dose of >10^6^ pfu MVA-HA-VN/04 prevented mortality caused by infection with both viruses.

**Table 2:** Survival after infection with A/VN/1194/04 or A/IND/5/05.

Number of vaccinations	Vaccine preparation	Dose(1)	A/VN/1194/04	A/IND/5/05
One	PBS	Mock	0/4	0/4
	wtMVA	10^8^	0/4	0/4
	MVA-HA-VN/04	10^3^	0/4	0/4
		10^4^	1/4	1/4
		10^5^	4/4	2/4
		10^6^	4/4	4/4
		10^8^	4/4	4/4
Two	PBS	Mock	0/4	0/4
	wtMVA	10^8^	0/4	0/4
	MVA-HA-VN/04	10^3^	2/3(2)	0/4
		10^4^	4/4	4/4
		10^5^	4/4	4/4
		10^6^	4/4	4/4

(1) Dose of MVA-HA-VN/04 in pfu (immunization in a total volume of 100 µl).

(2) One animal had to be euthanized before infection due to a complication unrelated to the experiment.

Two immunizations with 10^3^ pfu MVA-HA-VN/04 protected 67% of mice from death caused by infection with A/VN/04, but not that caused by infection with the heterologous strain A/IND/5/05 (Table 2). Two immunizations with a dose >10^4^ pfu of MVA-HA-VN/04, protected mice completely against mortality caused by infection with both influenza viruses.

### MVA-HA-VN/04 Vaccination Reduces Virus Replication in the Lungs

#### After one vaccination

**Figure 2:**
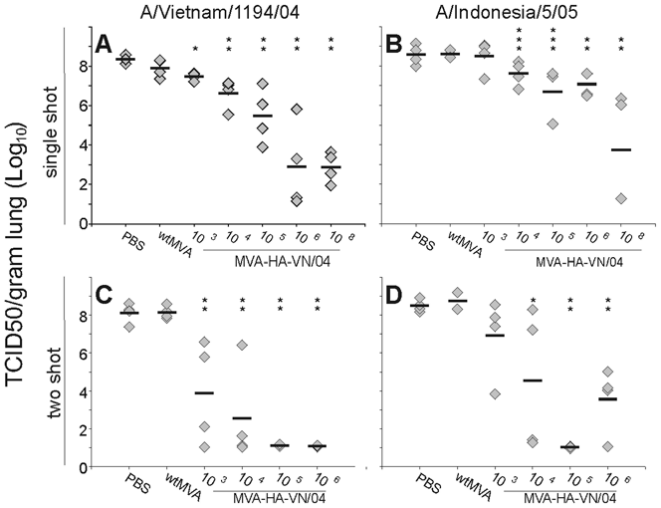
MVA-HA-VN/04 vaccination reduces virus replication in the lung. The data represent lung virus titers on day 4 post infection with influenza virus A/Vietnam/1194/04 and A/Indonesia/5/05 in mice that received one (A, B) or two (C, D) immunization(s) of: PBS, wtMVA or 10^3^, 10^4^, 10^5^, 10^6^ or 10^8^ (single shot only) pfu of MVA-HA-VN/04 as indicated. (* indicates a statistical significant difference with the PBS immunized group (p<0.05) (** indicates a significant difference with both the PBS and wtMVA immunized group (p<0.05)(*** indicates a statistical significant difference with the wtMVA immunized group).

Lungs were tested for the presence of infectious virus on day 4 post infection (p.i.). After infection with influenza virus A/VN/1194/04 of mice vaccinated with PBS or wtMVA, the mean virus titers were 10^8.3^ (SD = 10^0.2^) and 10^7.9^ (SD = 10^0.5^), respectively (Figure 2A). These titers were significantly higher than that of mice that were vaccinated with MVA-HA-VN/04 at a dose of 10^4^, 10^5^, 10^6^ or 10^8^ pfu (p<0.05). The mean virus titer in mice immunized once with 10^3^ pfu of MVA-HA-VN/04 was 10^7.5^ (SD = 10^0.2^), which was still significantly lower than that of mice that received PBS (p<0.05).

Four days post infection with influenza virus A/IND/5/05 the mean virus titers in the PBS vaccinated and wtMVA vaccinated mice were 10^8.5^ (SD = 10^0.6^) and 10^8.7^ (SD = 10^0.1^) respectively (Figure 2B). Mice vaccinated with a dose of 10^3^ pfu MVA-HA-VN/04 had similar mean virus titer of 10^8.5^ (SD = 10^0.8^). Vaccination with higher doses of MVA-HA-VN/04 resulted in lower lung virus titers. The extent of virus replication was vaccine dose dependent. The mean A/IND/5/05 virus titers for mice vaccinated with 10^4^, 10^5^, 10^6^ or 10^8^ pfu were 10^7.6^ (SD = 10^0.6^), 10^6.7^ (SD = 10^1.4^), 10^7.1^ (SD = 10^0.6^) and 10^3.7^ (SD = 10^2.8^), respectively.

#### After two vaccinations

As shown in figure 2C, two immunizations with MVA-HA-VN/04 resulted in significant lower lung virus titers four days after infection with the homologous virus A/VN/1194/04 compared to the PBS or empty vector inoculated mice, regardless the vaccine dose that was used. In mice vaccinated with vaccine doses >10^5^, infectious virus was not detected.

Four days p.i. with influenza virus A/IND/5/05 infectious virus could not be detected in the lungs of mice that were vaccinated twice with 10^5^ pfu of MVA-HA-VN/04. In mice vaccinated twice with 10^6^ pfu the mean virus titer in the lungs was 10^3.6^ (SD = 10^1.7^), which was significantly lower than that in the PBS and wtMVA immunized control mice which had mean titers of 10^8.5^ (SD = 10^0.3^) and 10^8.8^ (SD = 10^0.6^) respectively (Figure 2D). Also vaccination with a dose of 10^4^ pfu of MVA-HA-VN/04 significantly reduced the virus titers of A/IND/5/05 compared to PBS control mice.

### Vaccination Prevents Histopathological Changes in the Lungs after Influenza A/H5N1 Infection

**Figure 3:**
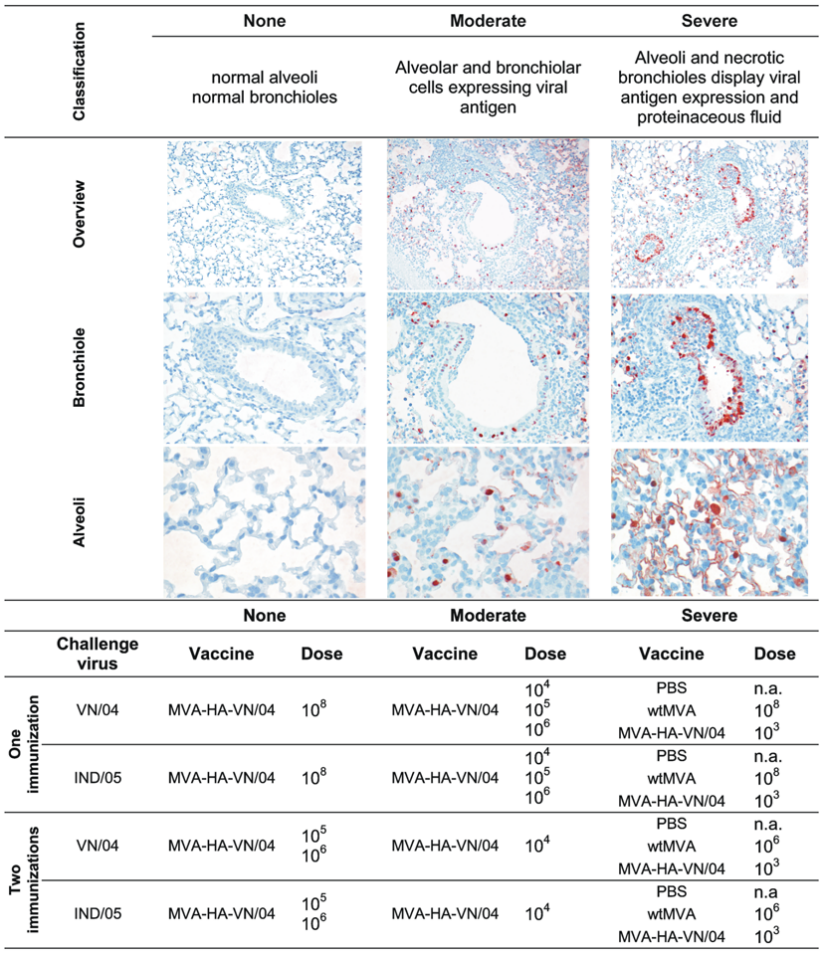
MVA-HA-VN/04 vaccination reduces histopathological changes induced by infection. Histopathological changes and the detection of virus-infected cells by immunohistochemistry in the lungs 4 days after infection with influenza A/H5N1 viruses A/VN/1194/04 and A/IND/5/05. Representative pictures were selected for the different classifications. Magnification: overview (10×), bronchiole (20×), alveoli (40×).

Upon infection with influenza viruses A/VN/1194/04 and A/IND/5/05, unprotected control mice inoculated with PBS or empty vector developed a moderate to severe broncho-interstitial pneumonia within four days (Figure 3). Histopathological changes were located in multifocal to coalescing lesions with more than 50% of the lungs affected. The lesions were characterized by marked inflammatory peribronchiolar lymphocytic infiltrates and occasionally proteinaceous fluid. There was necrosis in the bronchiolar epithelium resulting in cellular debri in the lumen. All these histopathological changes were located in multifocal to coalescing lesions with more than 50% of the lung affected. Similar lesions were observed in mice vaccinated with 10^3^ pfu of MVA-HA-VN/04. Mice that were immunized once with 10^4^, 10^5^ or 10^6^ pfu of MVA-HA-VN/04, or twice with 10^4^ pfu were partially protected against homologous and heterologous challenge infection. They displayed moderate changes in the lung: moderate peribronchiolar lymphocytic infiltrate and mild necrosis in the bronchiolar walls (Figure 3). Fourteen days p.i. normal architecture of the lung was restored in animals from these groups, apart from some residual peribronchiolar lymphocytic infiltrate and mild hyperplasia and hypertrophy of the bronchiolar and alveolar epithelium, consistent with regeneration. Mice vaccinated once with 10^8^ pfu of MVA-HA-VN/04 or twice with >10^5^ pfu displayed virtually no histopathological changes in the lung four days p.i. (Figure 3) or at later time points p.i. with influenza viruses A/VN/1194/04 or A/IND/5/05.

### Detection of Virus-Infected Cells by Immuno-Histochemistry

The presence of influenza virus-infected cells in the lungs was detected using a monoclonal antibody against the viral nucleoprotein, resulting in a red-brown precipitate in the nucleus and less in the cytoplasm. Four days p.i. with influenza virus A/VN/1194/04 infected cells were abundantly present in the lungs of control mice that received PBS or empty vector once or twice or mice vaccinated with 10^3^ pfu of MVA-HA-VN/04. Also after infection with influenza virus A/IND/5/05, virus-infected cells were abundantly present in the lungs of the control mice. A single vaccination with 10^3^, 10^4^, 10^5^ or 10^6^ pfu of MVA-HA-VN/04 did not prevent replication of influenza virus A/IND/5/05. The antigen-expressing cells were epithelial cells in the alveolar wall (type I and type II like pneumocytes) and bronchiolar epithelial cells in most of the bronchiolar walls (Figure 3). Mice vaccinated with 10^4^, 10^5^ or 10^6^ pfu of MVA-HA-VN/04 once or twice with 10^3^ pfu were partially protected against the homologous virus and had virus-infected cells, predominantly type II like pneumocytes, at multiple foci in their lungs (Figure 3). A few single infected cells were detected in the lungs of animals that had received a single immunization with 10^8^ pfu MVA-HA-VN/04 and virus-infected cells were virtually absent in animals that had received two immunizations with >10^5^ pfu of MVA-HA-VN/04 (Figure 3). No virus was detectable in any of the animals fourteen days after infection.

## Discussion

In the present study, the minimal requirements were assessed for the induction of protective immunity in mice against antigenically distinct influenza A/H5N1 viruses with a recombinant MVA expressing the HA gene of a clade 1 influenza A/H5N1virus. Two immunizations with a dose as low as 10^4^ pfu of MVA-HA-VN/04 were sufficient for the induction of protective immunity not only against the homologous strain but also against the antigenically distinct strain A/IND/5/05 from clade 2.1.

A dose of 10^4^ pfu is 10.000 fold lower than the dose of 10^8^ pfu that was used in previous studies that demonstrated the protective potential of MVA-HA-VN/04 vaccine candidate in mice and macaques [24], [25]. Thus, substantial less vaccine preparation is needed for the induction of protective immunity in mice. The possibility of dose-sparing would increase the number of individuals that can be vaccinated, with any amount of vaccine preparation, considerably. It was indicated on the website of a manufacturer of MVA based vaccines (www.bavarian-nordic.com) that the production capacity would range in tens of millions of doses, assuming a dose of 10^8^ pfu. If it can be confirmed that also in humans a dose of 10^4^ pfu is still effective, enough vaccine doses can be made for a global vaccination campaign. Thus the problem of the envisaged shortage of pandemic influenza vaccines could be addressed with the use of the recombinant MVA technology.

Another important issue that complicates the development of effective H5N1 vaccines is the co-circulation of antigenically distinct viruses. At present, nine different clades and subclades of A/H5N1 viruses have been identified and ideally vaccines will induce protective immunity against viruses of multiple clades. Two immunizations with MVA-HA-VN/04 afforded cross-clade protection. It should be noted that also protective effects were observed with low doses MVA-HA-VN/04 in the absence of detectable antibody responses specific for the two viruses used for challenge infection. It is possible that when low doses of vaccine are used antibody responses are induced below the detection limit, but which still afford some level of protection. Alternatively, it is possible that vaccination with low doses MVA-HA-VN/04 primed for secondary antibody responses or induced HA-specific T cell responses, which could have contributed to the protective effect. With higher doses of MVA-HA-VN/04 detectable antibodies were induced which indicated that the magnitude of the antibody responses is dependent on the vaccine dose.

In our mouse model, vaccination with two doses of ≥10^4^ pfu of MVA-HA-VN/04 reduced virus replication upon challenge infection significantly, which correlated with a reduction of histopathological changes in the lung and a reduction in the presentation of clinical signs, such as weight loss. Only with a high dose of MVA-HA-VN/04 (≥10^5^ pfu) sterilizing immunity was induced against the homologous strain. In some mice vaccinated twice with 10^6^ pfu of MVA-HA-VN/04, residual replication of influenza virus A/IND/5/05 was observed. However, this did not result in significant differences in clinical endpoints with the group of mice vaccinated with 10^5^ pfu, in which virus replication was not detected.

When a pandemic is imminent the rapid induction of protective immunity by vaccination is desirable and may prevent morbidity and mortality in selected population groups like health care workers or those at high risk for complications associated with infection with influenza viruses. Under these circumstances the instant induction of protective virus-specific immune responses by a single immunization without the need for a booster vaccination would be ideal. In the present study, we showed that a single immunization with MVA-HA-VN/04 protected mice from severe disease caused by infection with the homologous strain or the A/Indonesia/5/05 strain, especially when a high dose (10^8^ pfu) was used. However, also vaccination with lower doses in the range of 10^5^–10^6^ pfu afforded clinical protection, in particular against the homologous strain. In contrast to two immunizations, it was not possible to induce sterilizing immunity after a single immunization with MVA-HA-VN/04.

Collectively, we conclude that in addition to well-established favorable properties of MVA based vaccines such as superior safety, its good stability allowing stock-piling, high expression of genes of interest and good immunogenicity without the use of adjuvants, they also allow dose sparing. Two immunizations with relatively low doses of MVA-HA-VN/04 induced protective immunity against H5N1 viruses derived from different antigenically distinct clades. This vaccination strategy would be attractive for prepandemic vaccination, when there is still enough time for prime-boost regimens. Since there could be uncertainty about the strain that ultimately would cause a pandemic, the possibility to induce cross-clade immunity may afford broad protective immunity against a variety of different strains. When the induction of protective immunity becomes more urgent, a single immunization with a high dose might afford rapid protection against infection with the emerging pandemic strain. Of course the minimal requirements for the induction of protective immunity by MVA-HA-VN/04 vaccination need to be confirmed in humans. However, the potential of recombinant MVA-H5 vaccine was confirmed in non-human primates [24] and therefore we anticipate that also in humans dose sparing and single shot regimens are feasible. In this respect the presence of anti-vector immunity is considered to be a potential draw back of MVA based vaccines. Indeed it was demonstrated that pre-existing immunity to the vector especially affected T cell responses. This limitation could be overcome by mucosal administration of the vaccine or by using prime-boost regimens [34], [35]. However, since MVA is fully replication deficient, pre-existing immunity is unlikely to affect the immunogenicity of these vector vaccines to a great extent and did not prevent the induction of humoral responses to the expressed protein [35]–[37]. Thus recombinant MVA is promising as a H5N1 vaccine candidate, but of course this technology can be applied to other subtypes of influenza viruses as well. For example, it would be of interest to evaluate its potential as candidate vaccine against the pandemic influenza A/H1N1 virus that spread worldwide within two months, causing more than 52,000 reported cases, including over 231 deaths as of June 22^nd^ 2009 [2].

## Materials and Methods

### Ethics Statement

The experimental protocol was approved by Stichting Dier Experimenten Commissie (DEC) Consult before the start of the experiments, which were conducted according to national and international guidelines.

### Vaccine Preparation

Recombinant MVA expressing the HA gene of influenza virus A/Vietnam/1194/04 (MVA-HA-VN/04) was prepared as described previously [25]. MVA clonal isolate F6 served as the parental MVA virus. To generate final vaccine preparations, the virus was amplified in chicken embryo fibroblasts (CEF), purified by ultracentrifugation through sucrose, reconstituted in 1 mM Tris-HCL pH 9.0 and diluted in PBS.

### Viruses

Influenza viruses A/Vietnam/1194/04 (A/VN/1194/04) and A/Indonesia/5/05 (A/IND/5/05) were cultured in Madin Darby Canine Kidney (MDCK) cells. Infectious virus titers were determined in MDCK cells as described previously [38].

### Animals

Female specified pathogen free 6–8 weeks old C57BL/6J mice were purchased from Charles River (Sulzfeld, Germany) and were age-matched at the time point of the first immunization. Mice were immunized once with MVA-HA-VN/04 at a dose of 10^3^, 10^4^, 10^5^, 10^6^, or 10^8^ pfu in a volume of 100 µl intramuscularly in the hind legs (20 mice per dose). A second group of animals was immunized twice with MVA-HA-VN/04 at a dose of 10^3^, 10^4^, 10^5^, or 10^6^ pfu (20 mice per dose) with a time interval of four weeks. For the control groups mice were immunized with wildtype MVA (wtMVA) (10^6^ (two shot) or 10^8^ pfu (single shot)) (n = 60) or PBS (n = 56). Four weeks after the last immunization blood was drawn from the animals and they were infected with 10^3^ TCID_50_ of the homologous influenza virus A/VN/1194/04 or 10^3^ TCID_50_ of the heterologous influenza virus A/IND/05/05. Virus was inoculated intranasally in a volume of 50 µl and the challenge dose was chosen since it resulted in a lethal infection in >90% mice reproducibly (data not shown). Four and fourteen days after challenge infection mice were euthanized and their lungs were resected. Blood sampling, the intranasal infection, and euthanasia were carried out under anesthesia with inhalative isoflurane. The animals were housed in individual ventilated cage units (IVC-units) and had access to food and water *ad libitum*. During the infection with the influenza A/H5N1 viruses, animals were housed in type 3 cages placed in bio-safety level 3 containment facilities.

### Serology

After treatment with cholera filtrate and heat-inactivation at 56°C, the sera were tested for the presence of anti-HA antibodies. For this purpose a hemagglutination inhibition (HI) assay was used following a standard protocol using 1% turkey erythrocytes and four HA-units of influenza virus A/VN/1194/04 and A/IND/5/05 [39]. For this purpose reverse genetics viruses were produced from which the basic cleavage site in the HA molecule was deleted. The antibody titers obtained with these viruses were comparable with those obtained with the wild type strains (data not shown).

Sera were also tested for the presence of virus neutralizing antibodies specific for the two influenza viruses using a micro virus neutralization (VN) assay with the viruses that were produced by reverse genetics as described above [40]. In brief, 50 µl volumes of serial diluted serum samples were incubated with 100 TCID_50_ of the viruses for one hour at 37°C and then the mixture was added to MDCK cells. After one hour, the cells were washed and subsequently cultured in Eagles Minimal Essential Medium containing bovine serum albumin (BSA, fraction V 0.3%), 4 µg/ml trypsin, L-glutamin 2 mM, penicillin 100 U/ml, streptomycin 100 µg/ml NaHCO_3_ 0.15%, Hepes 20 mM and non-essential amino acids 0.1 mM. After five days, residual virus replication was assessed by measuring HA activity in the culture supernatants.

Hyper-immune serum obtained from a swan immunized twice with inactivated H5N2 influenza virus A/Duck/Potsdam/1402/86 (Nobilis influenza® H5N2 Intervet International, Boxmeer, the Netherlands) was used as a positive control against the two influenza viruses. For calculation purposes serum samples with an antibody titer of <10 were arbitrarily assigned a titer of 5.

### Lung Virus Titers

Lungs were snap frozen on dry ice with ethanol and stored at −70°C. Subsequently they were homogenized with a FastPrep-24® (MP Biomedicals, Eindhoven, The Netherlands) in transport medium (Hanks medium (MEM), lactalbumin, glycerol, penicillin, streptomycin, polymyxin B, nystatin, gentamicin) and centrifuged briefly. Quintuplicate ten-fold serial dilution of these samples were used to determine the virus titers on confluent layers of MDCK cells as described previously [38].

### Histopathology and Immunohistochemistry

Formalin-inflated lungs (two mice per group) were fixed in 10% neutral buffered formalin and then cross-sections were made and embedded in paraffin, sectioned at 4 µm and stained with hematoxylin and eosin for histological evaluation. Sequential slides were stained using an immunoperoxidase method with a monoclonal antibody (Clone HB65 IgG2a (American Type Culture Collection)) directed against the nucleoprotein of influenza A virus. a Goat-anti-mouse IgG2a HRP (Southern Biotech, Birmingham, Alabama, USA) was used as secondary antibody. The peroxidase was revealed using diamino-benzidine as a substrate, resulting in a deep red precipitate in the nuclei of influenza A virus-infected cells and a less intense red-staining of their cytoplasm. The sections were counterstained with hematoxylin.

### Statistical Analysis

Data for weight loss and viral titers were analyzed using the two-sided Student's *t* test and differences were considered significant at *P*<0.05.
